# Efficacy of SGLT2 inhibitors in preventing end-stage kidney disease in patients with IgA nephropathy: a scoping review

**DOI:** 10.3389/fmed.2026.1812783

**Published:** 2026-04-30

**Authors:** Atthaphong Phongphithakchai, Wiyada Kwanhian Klangbud, Thatsaphan Srithongkul, Sukit Raksasuk, Moragot Chatatikun

**Affiliations:** 1 Nephrology Unit, Division of Internal Medicine, Faculty of Medicine, Prince of Songkla University, Songkhla, Thailand; 2 Medical Technology Program, Faculty of Science, Nakhon Phanom University, Nakhon Phanom, Thailand; 3 Division of Nephrology, Department of Medicine, Faculty of Medicine, Siriraj Hospital, Mahidol University, Bangkok, Thailand; 4 Department of Medical Technology, School of Allied Health Sciences, Walailak University, Thasala, Nakhon Si Thammarat, Thailand

**Keywords:** eGFR slope, IgA nephropathy, kidney failure, proteinuria, SGLT2 inhibitors

## Abstract

**Objectives:**

To conduct a scoping review mapping the extent, nature, and consistency of available evidence regarding the effects of sodium–glucose cotransporter-2 (SGLT2) inhibitors on kidney function, proteinuria, kidney outcomes, and mortality in patients with IgA nephropathy (IgAN).

**Methods:**

This scoping review was conducted in accordance with the PRISMA-ScR guidance. Randomized controlled trials (RCTs) including patients with IgAN were identified to characterize high-level efficacy signals. Observational studies, real-world cohorts, and mechanistic investigations were additionally included to contextualize and extend the randomized evidence. Findings were mapped across five predefined outcome domains: acute eGFR dip, chronic eGFR slope, proteinuria reduction, kidney outcomes, and mortality.

**Results:**

Two large randomized controlled trials, DAPA-CKD and EMPA-KIDNEY, provided randomized evidence supporting kidney-protective effects of SGLT2 inhibitors in IgAN subgroups. Complementary observational and real-world studies consistently demonstrated clinically meaningful reductions in proteinuria, stabilization of kidney function trajectories, and mechanistic patterns linking early hemodynamic responses with longer-term renal benefit. Mortality data specific to IgAN remain limited; however, indirect evidence from broader CKD populations suggests biological plausibility for cardiovascular and survival benefits.

**Conclusion:**

The available evidence supporting SGLT2 inhibitors in IgA nephropathy is broad, convergent across study designs, and mechanistically coherent. Although disease-specific randomized trials remain limited, consistent signals from randomized, observational, and mechanistic evidence support the role of SGLT2 inhibitors as kidney-protective therapy in IgAN and highlight priorities for future IgAN-focused research.

**Systematic review registration:**

https://www.crd.york.ac.uk/prospero/display_record.php?RecordID=630522, identifier (CRD42025630522).

## 1 Introduction

Immunoglobulin A nephropathy (IgAN), also known as Berger’s disease, represents the most common form of primary glomerulonephritis worldwide, with particularly high prevalence in East and Southeast Asia (1). It is characterized by the mesangial deposition of polymeric IgA1-containing immune complexes, which lead to chronic glomerular inflammation, progressive glomerulosclerosis, and tubulointerstitial fibrosis (2). The clinical presentation is heterogeneous, ranging from isolated hematuria to full nephrotic syndrome and rapidly progressive glomerulonephritis (1, 2). Despite its relatively indolent presentation in many cases, IgAN carries a significant risk of progressive kidney function decline. Long-term cohort studies have reported that approximately 20–40% of patients eventually progress to end-stage kidney disease (ESKD) within 20–30 years of diagnosis (3). This disease burden is particularly troubling in low- and middle-income countries, where access to kidney replacement therapy may be limited. Thus, effective therapeutic strategies to delay or prevent progression to ESKD are urgently needed (4).

Management of IgAN primarily focuses on supportive care, including blood pressure control and renin-angiotensin system (RAS) blockade, which has proven effective in reducing proteinuria and slowing eGFR decline (5). The Kidney Disease: Improving Global Outcomes (KDIGO) guidelines recommend this as first-line therapy, particularly for patients with persistent proteinuria ≥0.5–1 g/day (6). Immunosuppressive agents, such as corticosteroids, may be considered in high-risk cases; however, randomized trials including TESTING and STOP-IgAN have raised concerns regarding their safety and long-term efficacy (7, 8).

Over the past decade, sodium-glucose co-transporter-2 (SGLT2) inhibitors have transformed the landscape of cardio-renal protection. Initially developed as glucose-lowering agents for type 2 diabetes mellitus, they have demonstrated a range of pleiotropic effects including natriuresis, improved tubuloglomerular feedback, reduction in intraglomerular pressure, anti-inflammatory effects, and amelioration of renal hypoxia that contribute to slowing the progression of chronic kidney disease (CKD), regardless of glycemic status (9). Landmark trials such as DAPA-CKD and EMPA-KIDNEY have demonstrated the renal benefits of SGLT2 inhibitors in both diabetic and non-diabetic CKD (10, 11). In DAPA-CKD, a pre-specified IgAN subgroup showed a significant reduction in sustained eGFR decline, ESKD, or renal death with dapagliflozin (10). These findings have increased interest in SGLT2 inhibitors as disease-modifying agents in IgAN. EMPA-KIDNEY further supported this by showing renal protection with empagliflozin in patients with primary glomerular diseases, including IgAN (11).

Evidence specific to IgAN, however, is distributed across prespecified subgroup analyses of large CKD trials, post-hoc analyses, and real-world observational studies. Given this heterogeneous evidence base and the limited number of disease-specific randomized trials, a scoping review approach is well suited to map the available evidence, clarify outcome domains studied, and identify knowledge gaps.

## 2 Methods

### 2.1 Protocol registration

We conducted this study as a scoping review in accordance with the Joanna Briggs Institute (JBI) methodology for scoping reviews and reported the findings following the Preferred Reporting Items for Systematic Reviews and Meta-Analyses Extension for Scoping Reviews (PRISMA-ScR) guidelines (12). The PRISMA-ScR checklist is provided in Supplementary Table 1. The primary objective of this scoping review was to map the extent, range, and nature of available evidence regarding the use of SGLT2 inhibitors in patients with IgA nephropathy, rather than to estimate pooled treatment effects or comparative efficacy. The review protocol was prospectively registered in the International Prospective Register of Systematic Reviews (PROSPERO) (Registration No. CRD42025630522).

### 2.2 Eligibility criteria

Eligibility criteria were defined according to the participant-concept-context framework recommended for scoping reviews. Participants were adult patients who were aged 18 years or older and had biopsy-confirmed IgA nephropathy; the concept of interest was the use of SGLT2 inhibitors, including dapagliflozin, empagliflozin, or canagliflozin; and the context encompassed clinical studies evaluating kidney-related outcomes in IgA nephropathy. Eligible evidence sources included randomized controlled trials (RCTs) and observational studies (prospective or retrospective cohorts and real-world studies). Studies were required to report at least one kidney-related outcome, such as changes in eGFR, proteinuria, kidney disease progression, initiation of chronic dialysis, kidney transplantation, or kidney-related death. We excluded reviews, editorials, letters, commentaries, conference abstracts without full text, expert opinions, case reports, case series, animal studies, and *in vitro* or *ex vivo* studies. Articles not published in English or published after December 2024 were also excluded.

### 2.3 Search strategy

A comprehensive literature search was performed in PubMed/MEDLINE, Embase, and Web of Science databases through December 2024. The search strategy combined terms related to the population (“IgA nephropathy,” “Berger’s disease,” “primary glomerulonephritis”), interventions (“SGLT2 inhibitor,” “sodium glucose co-transporter 2 inhibitor,” “dapagliflozin,” “empagliflozin,” “canagliflozin”), and outcomes (“eGFR,” “ESKD,” “chronic dialysis,” “renal transplantation,” “death”), along with study design filters (“randomized controlled trial,” “observational study”). Detailed search strings for each database are provided in Supplementary Table 2. Additional records were identified through manual screening of reference lists.

### 2.4 Study selection and data extraction

All identified records were imported into Zotero reference management software, and duplicate citations were removed. Titles and abstracts were independently screened by two reviewers (A. P. and M. C.) for eligibility. Full-text articles of potentially relevant studies were subsequently reviewed in detail against the inclusion criteria by the same reviewers. Any disagreements at either stage were resolved through discussion, and when necessary, consultation with a third reviewer. The study selection process is summarized and presented in a PRISMA flow diagram. A standardized data-charting form was developed collaboratively by the review team. Two reviewers (A. P. and M. C.) independently extracted data from each included study. Extracted information included study design, sample size, patient population, type of SGLT2 inhibitor, follow-up duration, background therapies, and reported kidney-related outcomes. For randomized controlled trials, data were charted on trial design, baseline characteristics, and primary kidney outcomes. For observational and real-world studies, data were charted on study objectives, proteinuria outcomes, kidney function trajectories, and mechanistic endpoints. Outcomes of interest included acute changes in eGFR, chronic eGFR slope, proteinuria, kidney disease progression, and mortality.

## 3 Results

### 3.1 Literature search and selected studies

**Figure 1:**
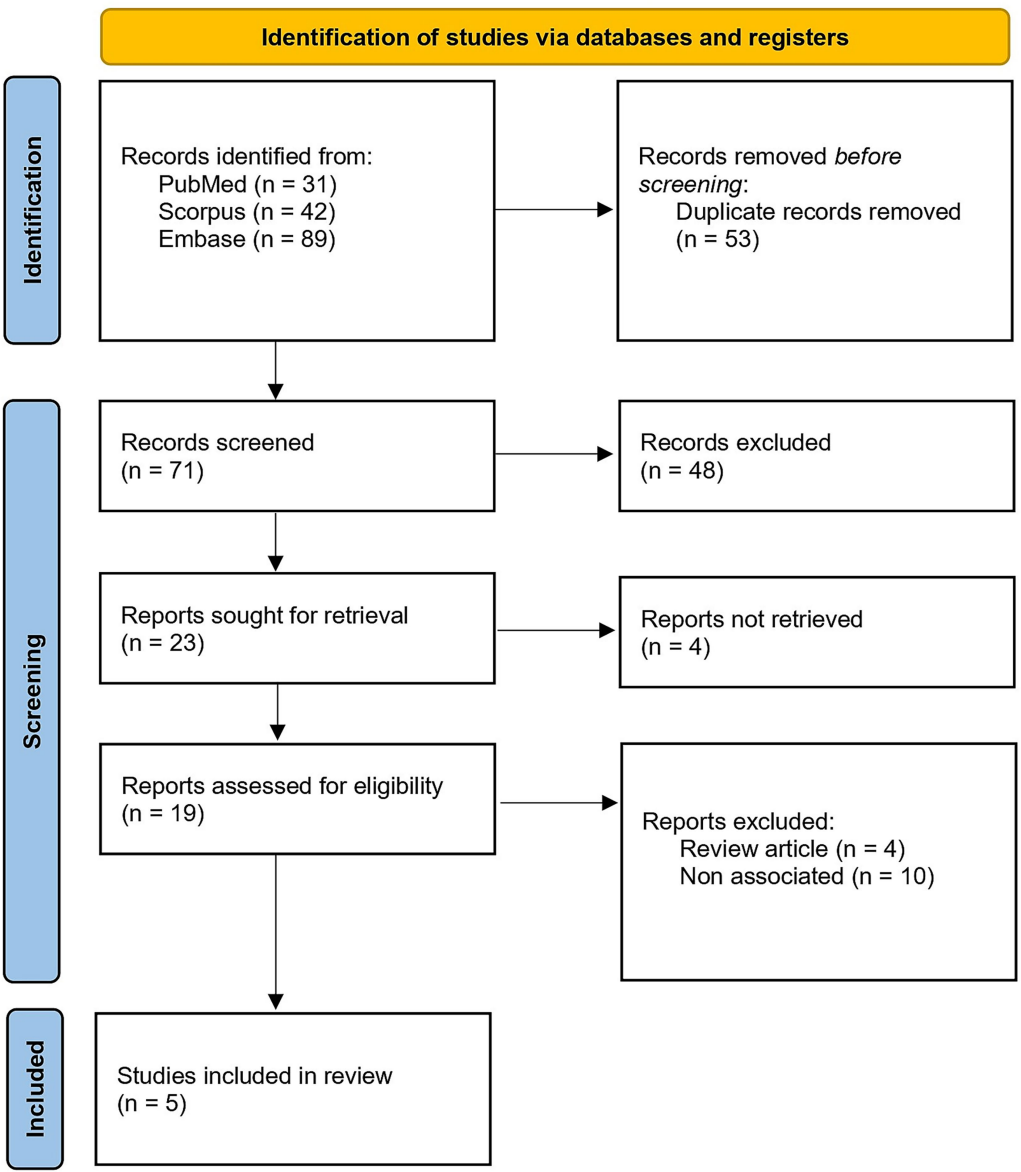
PRISMA flow diagram of study screening and selection.

A total of 166 records were identified through database searches in PubMed/MEDLINE (*n* = 31), Embase (*n* = 89), and Web of Science (*n* = 46). After removal of 53 duplicate records, 113 records remained for title and abstract screening. Of these, 90 records were excluded, and 23 full-text articles were assessed for eligibility. Four full-text articles could not be retrieved. Among the 19 full-text articles reviewed, 14 were excluded because they were review articles, editorials, or did not report relevant kidney-related outcomes in IgA nephropathy. Ultimately, two randomized controlled trials and three observational or real-world studies were included in this scoping review. The study selection process is summarized in the PRISMA flow diagram (Figure 1).

#### 3.1.1 Randomized controlled trials

The two included randomized controlled trials were (18) and EMPA-KIDNEY (2024), which together contributed 1,087 patients with IgA nephropathy (10, 11). Of these, 550 patients received SGLT2 inhibitors (dapagliflozin or empagliflozin) and 537 received placebo. In the DAPA-CKD trial, 137 patients in the IgA nephropathy subgroup received dapagliflozin and 133 received placebo; the mean baseline eGFR was 43.8 mL/min/1.73 m^2^, the median urinary albumin-to-creatinine ratio (UACR) was 900 mg/g, and the median follow-up duration was 2.1 years. In the EMPA-KIDNEY trial, 413 patients received empagliflozin and 404 received placebo; the mean baseline eGFR was 43.3 mL/min/1.73 m^2^, the median UACR was 662 mg/g, and the median follow-up was 2.0 years. Both trials evaluated kidney disease progression using composite renal or renal–cardiovascular outcomes as their primary endpoints. Key characteristics of the randomized trials are summarized in Table 1.

**Table 1:** Study characteristics of SGLT2 inhibitors in IgA nephropathy.

A. Randomized controlled trials									
Study	Design	Intervention	Control	Number of participants in intervention group	Number of participants in control group	eGFR, mean(SD), mL/min/1.73m^2^	UACR, median, mg/d	Follow-up	Primary outcome
DAPA-CKD (18)	RCT (pre specified subgroup analysis)	Dapagliflozin	Placebo	137	133	43.8	900	2.1 years	50% ↓ eGFR, ESKD, death from renal/CV cause
EMPA-KIDNEY (16)	RCT (secondary analysis)	Empagliflozin	Placebo	413	404	43.3	662	2.0 years	Kidney disease progression or CV death

B. Observational and real-world studies									
Study	Design	Population	Intervention	Number of patients	Baseline eGFR	Baseline proteinuria	Follow up	Primary objective	Secondary objective
Dong et al. (13)	Prospective cohort	Biopsy-proven IgAN on full-dose RAAS blockade	SGLT2 inhibitors	93 (62 completed 6 month)	Mean 55 mL/min/1.73m^2^	Median urine protein 1.32 g/24 h	6 months	Antiproteinuric effect in IgAN	Short-term eGFR change and safety
Caravaca-Fontán et al. (14) (GLOSEN)	Multicenter retrospective cohort	Primary and secondary GN (IgAN subgroup included)	SGLT2 inhibitors	493 total (IgAN 192)	Median 56 mL/min/1.73 m^2^	Median UACR 1,287 mg/g	12 months	Real-world antiproteinuric effectiveness	Proteinuria–eGFR slope association
Schork et al. (15)	Prospective observational	CKD included IgAN (31%)	SGLT2 inhibitors	42 (IgAN 13)	Median 46 mL/min/1.73 m^2^	Median UACR 1881 mg/g	6 months	Effect on volume status in CKD	Changes in albuminuria and kidney function

CKD; chronic kidney disease, CV; cardiovascular, ESKD; end-staged kidney disease, eGFR; estimated glomerular filtration rate, IgAN; immunoglobulin A nephropathy, SGLT2; sodium glucose cotransporter 2, UACR; urinary albumin creatinine ratio.

#### 3.1.2 Observational and real-world studies

Three observational or real-world studies were included to complement the randomized evidence. A prospective cohort study by Dong et al. enrolled patients with biopsy-proven IgA nephropathy receiving optimized RAAS blockade and evaluated the antiproteinuric effect of SGLT2 inhibitors over a 6-month follow-up (13). A multicenter retrospective cohort study from the GLOSEN group assessed the real-world effectiveness of SGLT2 inhibitors on proteinuria across primary and secondary glomerular diseases, including IgA nephropathy, with 12 months of follow-up (14). In addition, a prospective observational study by Schork et al. evaluated the effects of SGLT2 inhibitors on volume status and overhydration in patients with chronic kidney disease, including an IgA nephropathy subgroup, over 6 months (15). The design characteristics, populations, and study objectives of these observational studies are summarized in Table 1.

### 3.2 Mapping of evidence by outcome domain

To clarify how evidence from different study designs informs clinically relevant outcomes, findings were mapped across the five outcome domains. This structured mapping is presented in Table 2.

**Table 2:** Mapping of evidence for SGLT2 inhibitor effects in IgA nephropathy by outcome domain.

Outcome domain	RCTs	Key findings from RCTs	Observational or real-world evidence	Key supportive findings
Acute eGFR dip	DAPA-CKD, EMPA-KIDNEY	Early, modest decline in eGFR after initiation, stabilizing within weeks; not associated with long-term harm	CKD and glomerulonephritis cohorts	Acute dip consistently observed and interpreted as hemodynamic (restoration of tubuloglomerular feedback)
Chronic eGFR slope	DAPA-CKD, EMPA-KIDNEY	Slower long-term eGFR decline compared with placebo in IgAN subgroups	International GN cohorts and real-world CKD studies	Patients achieving proteinuria reduction showed significantly slower annual eGFR loss
Proteinuria reduction	DAPA-CKD (secondary outcome)	Reduction in albuminuria accompanying kidney protection	Biopsy-proven IgAN cohorts; multinational GN studies	20–30% reduction at 3–6 months in IgAN; up to 40–50% reduction across GN populations
Kidney outcomes (ESKD or renal composite)	DAPA-CKD, EMPA-KIDNEY	Reduced risk of kidney disease progression (≥40–50% eGFR decline, ESKD, renal death) with consistent effects across primary kidney diseases	Meta-analytic and real-world CKD data	Consistent reduction in kidney failure events irrespective of diabetes or kidney etiology
Mortality outcomes	DAPA-CKD (secondary); EMPA-KIDNEY	Reduction in all-cause and cardiovascular mortality in CKD populations	Long-term IgAN cohorts	IgAN associated with excess mortality vs. general population; risk highest with proteinuria and reduced eGFR

CKD, chronic kidney disease; ESKD, end-staged kidney stage; eGFR, estimated glomerular filtration rate; GN, glomerulonephritis; IgAN, immunoglobulin A nephropathy.

#### 3.2.1 Acute eGFR dip

Across randomized trials and observational cohorts, initiation of SGLT2 inhibitors was consistently associated with an early, modest decline in eGFR. Mechanistic analyses indicate that this phenomenon reflects restoration of tubuloglomerular feedback and reduction in intraglomerular pressure rather than structural kidney injury. Importantly, the acute dip stabilized within weeks and was not associated with adverse long-term kidney outcomes in large CKD trials.

#### 3.2.2 Proteinuria reduction

Proteinuria reduction emerged as the most consistent and robust outcome across study designs. In a biopsy-proven IgAN cohort receiving full-dose RAS blockade, SGLT2 inhibitor therapy reduced proteinuria by approximately 23% at 3 months and 27% at 6 months, independent of baseline eGFR, immunosuppressive use, or diabetes status. These findings were reinforced by large real-world studies of primary and secondary glomerulonephritis, which demonstrated sustained proteinuria reductions of 35–48% over 12 months, with greater reductions associated with higher baseline serum albumin levels.

#### 3.2.3 Chronic eGFR slope

Randomized trials demonstrated slower long-term eGFR decline with SGLT2 inhibitors across CKD populations, including patients with glomerular disease. Observational cohorts further showed that patients achieving greater proteinuria reduction experienced significantly slower annual eGFR loss, supporting proteinuria as a mediator linking early hemodynamic effects to durable kidney protection.

#### 3.2.4 Kidney outcomes (ESKD and renal composite endpoints)

In prespecified analyses of DAPA-CKD, dapagliflozin reduced the risk of sustained ≥50% eGFR decline, ESKD, or kidney-related death, with consistent effects observed in patients with glomerulonephritis. EMPA-KIDNEY extended these findings by demonstrating kidney protection irrespective of diabetes status or primary kidney diagnosis. Collaborative analyses of large placebo-controlled trials further demonstrated an approximately 37% reduction in kidney disease progression with SGLT2 inhibitors, with no evidence of effect modification by diabetes or underlying kidney disease category.

#### 3.2.5 Mortality outcomes

Direct evidence for mortality reduction in IgAN treated with SGLT2 inhibitors remains limited. Long-term observational studies demonstrate excess mortality in IgAN compared with the general population, particularly among patients with persistent proteinuria and reduced eGFR. In contrast, large CKD trials have shown reductions in cardiovascular and all-cause mortality with SGLT2 inhibitors, providing indirect but biologically coherent support for potential survival benefit.

## 4 Discussion

In this scoping review, we mapped the extent, nature, and consistency of evidence regarding SGLT2 inhibitors in biopsy-confirmed IgA nephropathy across five clinically relevant outcome domains: acute eGFR dip, chronic eGFR slope, proteinuria reduction, kidney outcomes, and mortality. The evidence base comprises two large, randomized trials that included IgAN subgroups (DAPA-CKD and EMPA-KIDNEY) and three observational/real-world studies that contextualize and extend the randomized signals. Across study designs, the overall pattern of findings is convergent: initiation of SGLT2 inhibitors is associated with a predictable early eGFR dip that stabilizes, consistent reductions in proteinuria over months, and slower longer-term eGFR decline. Evidence on hard kidney endpoints (e.g., ESKD) is derived primarily from composite outcomes in large CKD trials, whereas mortality data specific to IgAN remain limited and largely indirect.

IgA nephropathy is a leading cause of CKD and ESKD, and supportive care with blood pressure control and RAAS blockade remains the cornerstone of therapy. Recent advances in CKD therapeutics have highlighted SGLT2 inhibitors as kidney-protective agents across diabetic and non-diabetic CKD populations. Our mapping confirms that IgAN-specific randomized evidence largely arises from prespecified or secondary subgroup analyses within broad CKD trials rather than IgAN-dedicated randomized trials.

In DAPA-CKD, a prespecified analysis of patients with IgAN demonstrated a substantial reduction in the composite outcome of sustained eGFR decline, ESKD, or kidney-related death, supporting the plausibility of meaningful benefit in this disease. Similarly, EMPA-KIDNEY extended the generalizability of SGLT2 inhibitor kidney protection to a wide spectrum of CKD etiologies, including glomerular diseases, with consistent effects observed across primary kidney diagnoses. However, when interpreted specifically for IgAN, randomized evidence remains constrained by subgroup sample sizes, variability in endpoint definitions, and limited reporting of IgAN-specific granular outcomes (10, 11, 16).

Observational and real-world studies included in this review provide important complementary insights. In a prospective cohort of biopsy-proven IgAN patients receiving optimized RAAS blockade, Dong et al. reported that SGLT2 inhibitor therapy was associated with clinically meaningful reductions in proteinuria over a 6-month follow-up. Patients had a mean baseline eGFR of approximately 55 mL/min/1.73 m^2^ and median baseline proteinuria of 1.32 g/day, reflecting a population at moderate risk of progression (13). Similarly, Caravaca-Fontán et al., in a multicenter retrospective cohort from the GLOSEN group, evaluated 493 patients with primary and secondary glomerular diseases, including 192 patients with IgAN. In this real-world population (median baseline eGFR 56 mL/min/1.73 m^2^; median UACR 1,287 mg/g), SGLT2 inhibitor use was associated with significant and sustained proteinuria reductions over 12 months (14). In addition, Schork et al. demonstrated that SGLT2 inhibitors improved volume-related parameters in CKD patients, including an IgAN subgroup, with a median baseline eGFR of 46 mL/min/1.73 m^2^ and median UACR of 1,881 mg/g over 6 months of follow-up (15). Although observational studies are inherently susceptible to confounding and selection bias, the consistency of findings across cohorts particularly regarding proteinuria reduction and stabilization of kidney function supports external validity and biological plausibility of SGLT2 inhibitor effects in IgAN.

A notable contribution of this review is the integration of clinical outcomes with mechanistic plausibility. The early eGFR dip, observed consistently after SGLT2 inhibitor initiation, is best interpreted as a hemodynamic response reflecting restoration of tubuloglomerular feedback and reduction in intraglomerular pressure rather than structural kidney injury. This interpretation aligns with randomized trial observations of slower chronic eGFR decline and with observational evidence showing that patients achieving greater proteinuria reductions tend to exhibit slower annual eGFR loss. Together, these converging signals support a mechanistically coherent pathway linking early functional changes to longer-term kidney protection in IgAN.

Beyond hemodynamic effects, SGLT2 inhibitors may exert reno-protective actions through anti-inflammatory and anti-oxidative pathways. Preclinical studies suggest modulation of autophagy and klotho-related signaling, with restoration of impaired autophagy under hyperglycemic conditions. In addition, upregulation of *α*-klotho may contribute to anti-inflammatory and anti-fibrotic effects (17). Although derived from experimental models, these findings support the biological plausibility of SGLT2 inhibitor–mediated kidney protection. In IgA nephropathy, where progression involves inflammation and oxidative injury, these mechanisms may help explain the consistent antiproteinuric and eGFR-stabilizing effects observed in clinical studies.

From a clinical perspective, the mapped evidence supports consideration of SGLT2 inhibitors as an adjunct to optimized supportive care in IgAN, particularly among patients with persistent proteinuria despite maximized RAAS blockade. Across the included observational cohorts and real-world studies, proteinuria reduction emerged as the most consistent finding, with clinically meaningful decreases observed within 3–6 months and sustained reductions reported over 12 months in broader glomerular disease cohorts that included IgAN. These antiproteinuric effects are relevant because proteinuria is a key risk marker and mediator of progression in IgAN. In addition, clinicians should anticipate a modest early decline in eGFR after treatment initiation; this phenomenon is typically transient and stabilizes within weeks, and its presence should generally be interpreted in the context of expected hemodynamic effects rather than immediate treatment failure, provided no alternative cause of kidney injury is evident.

Nevertheless, this review also highlights the need for careful framing: current IgAN-specific randomized evidence is derived from subgroup analyses within large CKD trials, and hard endpoints such as ESKD and kidney-related death have not yet been tested in adequately powered IgAN-dedicated RCTs. Therefore, treatment decisions should integrate patient risk profile, background supportive therapy optimization, kidney function, and safety considerations, while acknowledging that the strongest efficacy signals come from broader CKD trial populations that included IgAN.

This review has several strengths. First, it provides a structured mapping of evidence across outcome domains that matter in IgAN clinical care, enabling readers to understand where evidence is strongest (proteinuria reduction, eGFR trajectory) and where gaps remain (IgAN-specific mortality and hard kidney endpoints). Second, by integrating randomized subgroup evidence with real-world and observational data, the review contextualizes efficacy signals and supports biological plausibility. Third, restricting key clinical trial evidence to IgAN populations within landmark CKD trials improves relevance for glomerular disease practice.

Several limitations should be acknowledged. The randomized evidence in IgAN remains limited to two trials with IgAN subgroups and differing endpoint definitions. In addition, important limitations related to study populations should be considered. In EMPA-KIDNEY, not all patients had biopsy-confirmed IgA nephropathy, as clinical diagnoses were also accepted, which may introduce misclassification and heterogeneity, particularly in the presence of diabetes. Differences in patient populations across trials further complicate interpretation. DAPA-CKD included patients with more advanced kidney disease and higher baseline proteinuria, whereas EMPA-KIDNEY enrolled a broader CKD population, including those with lower or absent proteinuria and a wider range of kidney function. These differences may affect both the magnitude and mechanisms of treatment effects and limit direct comparability. Potential differences between Asian and non-Asian populations should also be considered, given known geographic variability in disease phenotype, progression, and treatment response, which may affect generalizability. Observational studies provide supportive context but are subject to confounding and heterogeneity. As a scoping review, our aim was to map the evidence rather than perform quantitative synthesis. Finally, mortality outcomes specific to IgAN remain limited, and conclusions regarding survival benefit should be interpreted as indirect.

In summary, evidence supporting SGLT2 inhibitors in IgA nephropathy is broad and convergent across randomized subgroup analyses and observational/real-world studies. The collective findings are mechanistically coherent, with an expected early eGFR dip followed by stabilization, consistent proteinuria reductions, and signals of slower long-term eGFR decline and kidney disease progression. While IgAN-dedicated randomized trials remain limited, the mapped evidence supports the role of SGLT2 inhibitors as kidney-protective therapy in IgAN and highlights priorities for future IgAN-specific research.

## Data Availability

Publicly available datasets were analyzed in this study. These data are derived from sources cited within the article.
